# Lung abscess as a complication of COVID‐19 infection, a case report

**DOI:** 10.1002/ccr3.3686

**Published:** 2021-01-25

**Authors:** Nazanin Zamani, Oldooz Aloosh, Samane Ahsant, Zeynab Yassin, Aminreza Abkhoo, Taghi Riahi

**Affiliations:** ^1^ Department of Internal Medicine School of Medicine Hazrat‐ e Rasool General Hospital Iran University of Medical sciences(IUMS) Tehran Iran; ^2^ Department of Infectious Disease School of Medicine Hazrat‐ e Rasool General Hospital Iran University of Medical Sciences(IUMS) Tehran Iran; ^3^ Antimicrobial Resistance Research Center Iran University of Medical Sciences Tehran Iran; ^4^ Department of Radiology School of Medicine Imam Khomeini General Hospital Tehran University of Medical Sciences(TUMS) Tehran Iran

**Keywords:** antibiotic therapy, complication, COVID‐19, lung abscess

## Abstract

To our knowledge, no previous studies have reported lung abscess as a complication of COVID‐19 infection. It is essential to follow‐up with the patients after discharge for such complications, especially if they are symptomatic.

## Graphical abstract

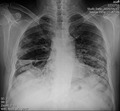

## 1 INTRODUCTION

A 53‐year‐old man was admitted to the hospital and was treated for COVID‐19. Twenty‐two days after discharge, the patient made an appointment complaining of persistent cough and hemoptysis. Follow‐up chest X‐ray showed an air‐fluid level in the right lung. The chest CT scan showed a lung abscess

**FIGURE 1:**
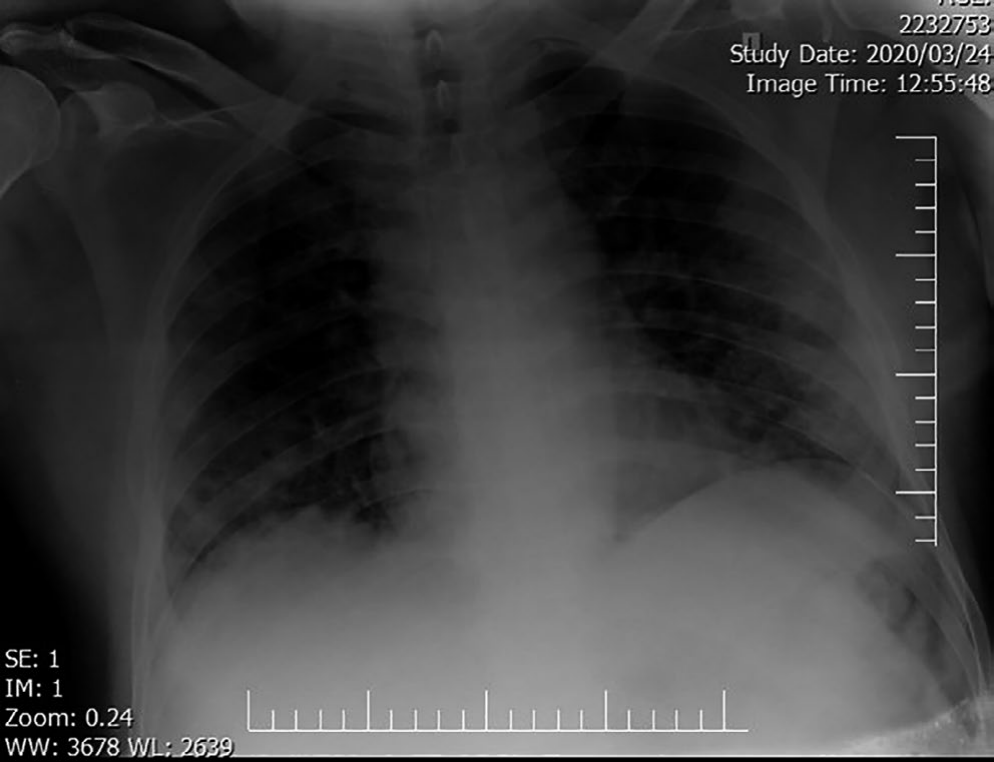
Chest X‐ray at first admission

**FIGURE 2:**
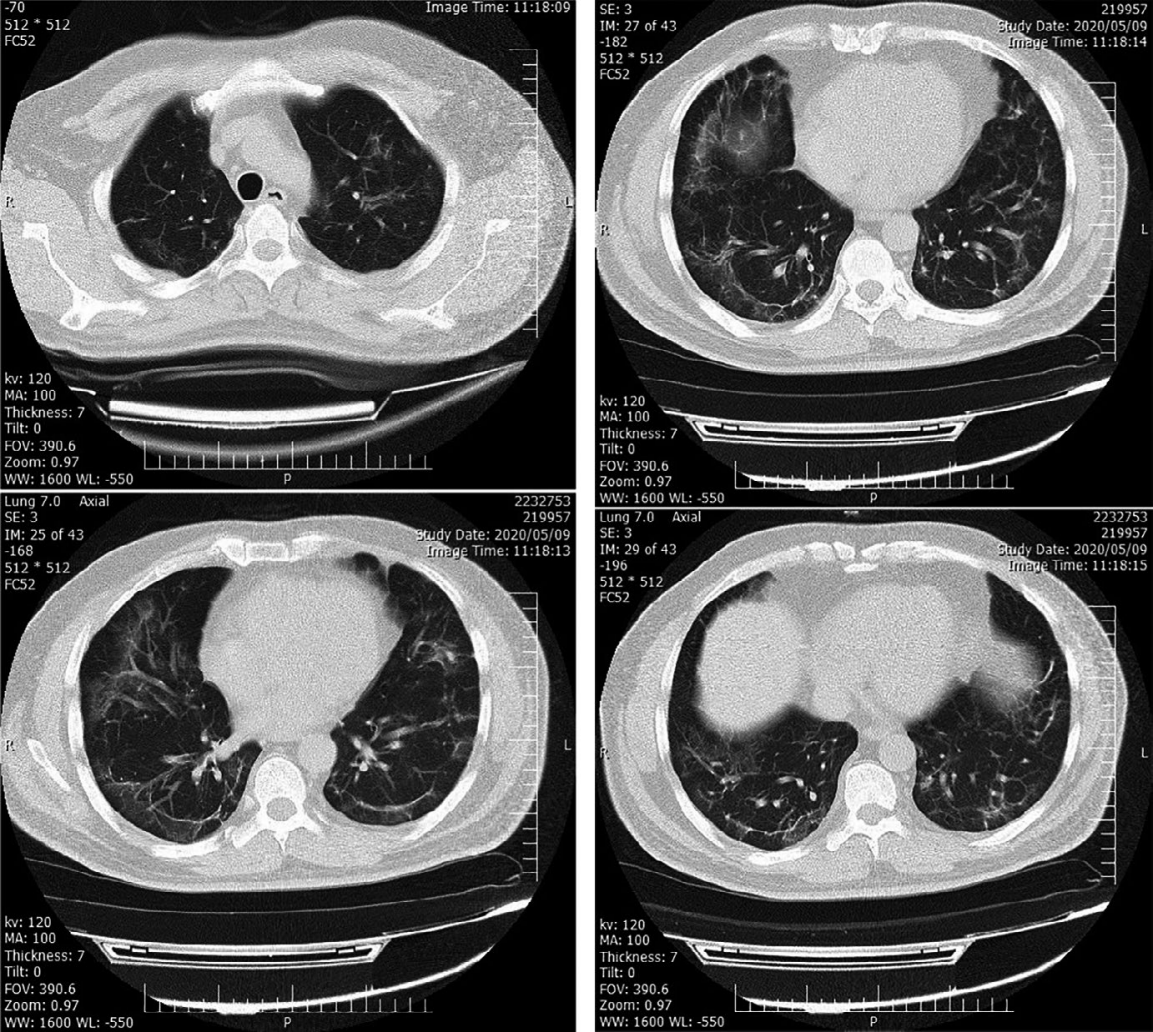
Chest CT scan at first admission

**FIGURE 3:**
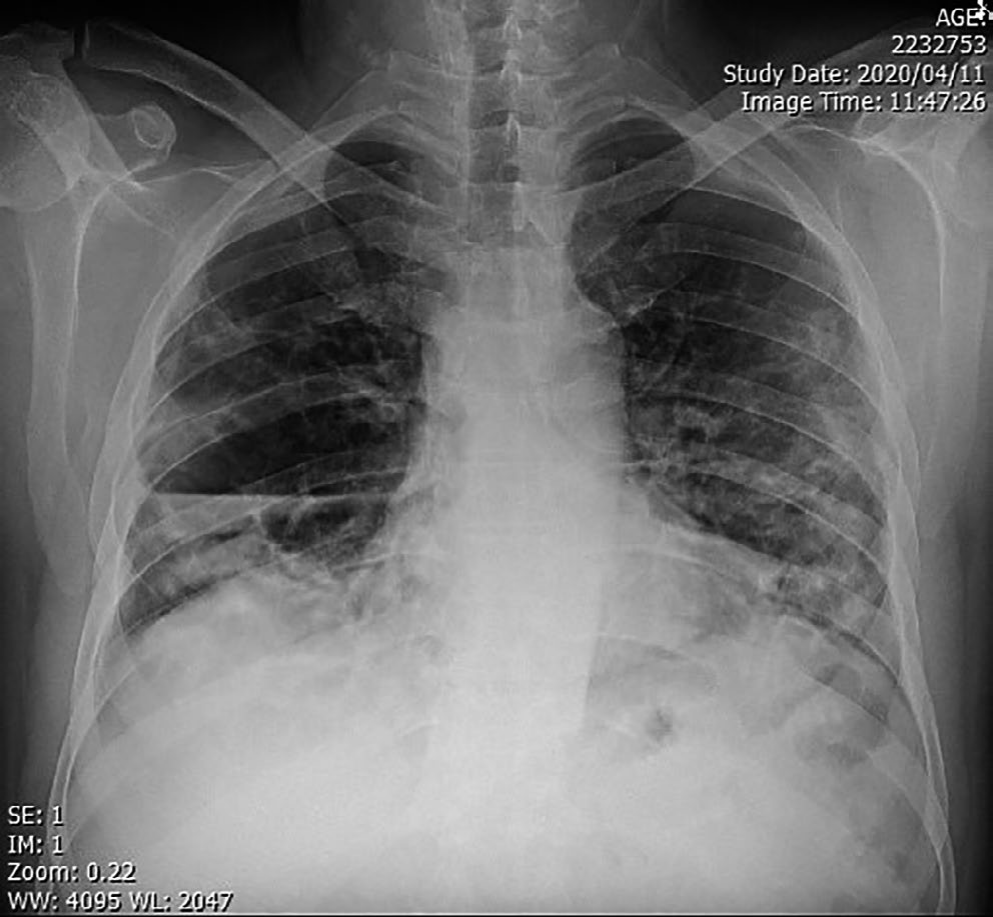
Chest X‐ray at second admission

**FIGURE 4:**
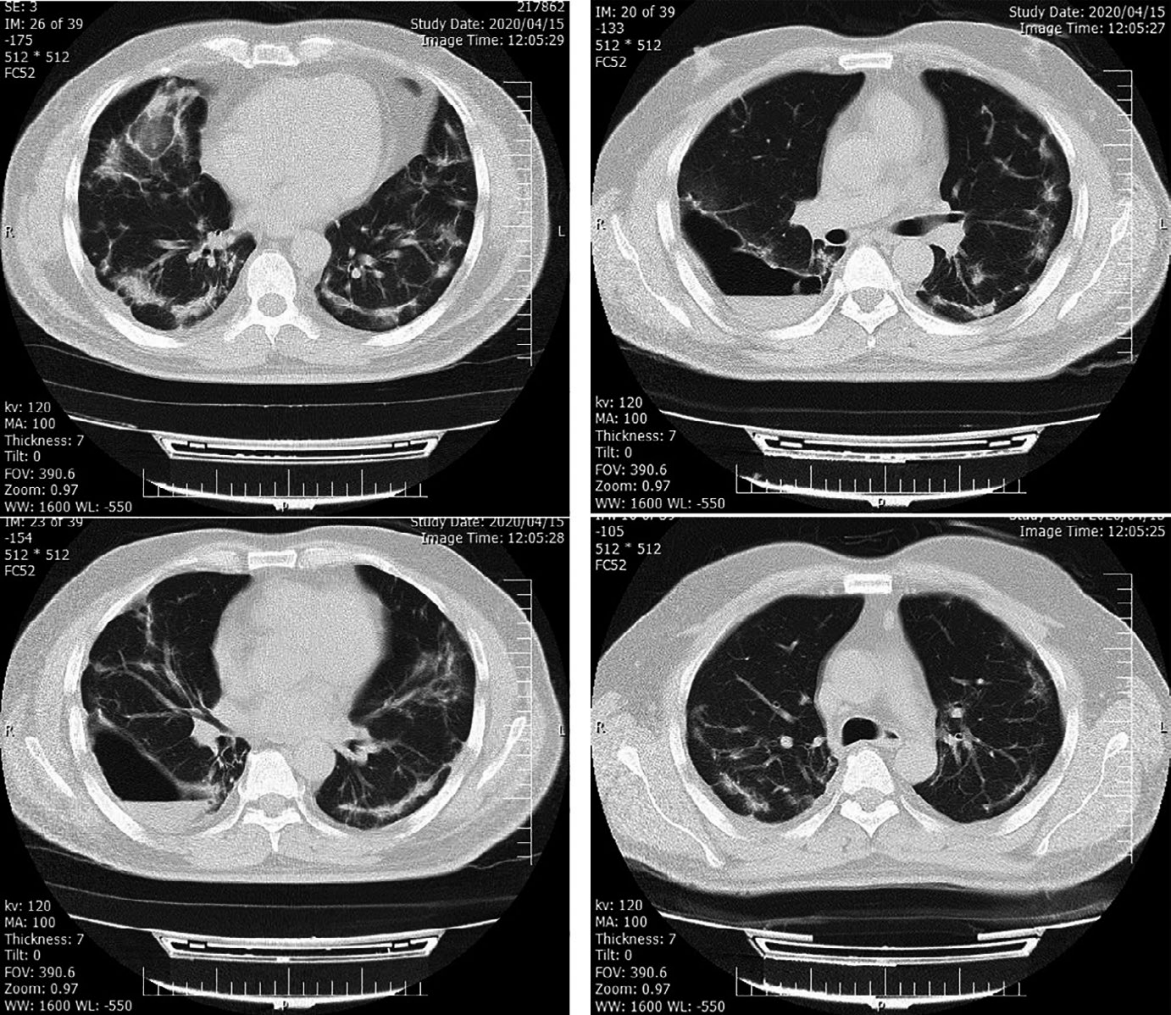
Chest CT scan with lung abscess

In late 2019, a new respiratory infection was spreading all around the world.^1^ Novel coronavirus disease (COVID‐19) became a pandemic according to the World Health Organization on 11 March 2020. The first COVID‐19 patient in Iran was diagnosed on 19 February 2020, at Qom city. In Hazrat‐e‐Rasoul general hospital, the first administered patient was diagnosed on 20 February 2020, after a short trip to Qom (Figures 1, 2, 3, 4).

The disease's main clinical characteristics are myalgia, fever, cough, respiratory distress, and shortness of breath.^2^, ^3^

The diagnosis of COVID‐19 is based on the clinical manifestations, laboratory testing, and imaging. Reverse transcription polymerase chain reaction (RT‐PCR) test from nasopharyngeal swab samples and typical chest computerized tomography (CT) characteristics confirms the diagnosis of COVID‐19.^4^ Chest CT scan had a sensitivity of 97% for diagnosing COVID‐19 based on physicians’ experience, which is even superior to RT‐PCR.^5^

Every day, a new manifestation of COVID‐19 is revealed. Late complications of COVID‐19 infection are not fully understood. The treatments and other interventions used for COVID‐19 are mostly experimental, and the effectiveness and complications of such treatments are still unknown. As medical workers, we must inform our coworkers and share our experiences. In this article, we are presenting a COVID‐19‐positive patient with remarkable complication after receiving treatment.

## 2 CASE PRESENTATION

A 53‐year‐old previously healthy man admitted to the isolated respiratory ward due to fever, myalgia, cough, and shortness of breath. On the initial examination, he had a low‐grade fever with a temperature of 37.8 centigrade and tachypnea with a respiratory rate of 23 per minute. Oxygen saturation was 90 percent on room air. His lungs examination revealed mild crackles in both basal lungs fields. Chest CT scan showed ground glass, patchy pleural‐based consolidations of lung parenchyma.

The patient was treated with Hydroxychloroquine 200mg every 12 hours, accompanied by other supportive cares such as hydration and oxygen therapy.

Four days after admission, the symptom began to recuperate, and the patient was discharged to receive the rest of the treatment course at home. The patient receives Hydroxychloroquine for 14 days. It was recommended that the patient visit the day clinic for a better follow‐up of the healing process.

The patient made an appointment to our day clinic 22 days after discharge, complaining of persistent cough and hemoptysis. Therefore, a follow‐up chest X‐ray was taken. To our surprise, an air‐fluid level was seen in the lower lobe of the right lung.

For a better understanding of the etiology of this imaging finding, a lung chest CT scan was ordered, and the patient was admitted to an isolated respiratory ward for further evaluation and treatment. The lung CT scan showed an abscess with fluid in the right lower lobe. The patient was treated with intravenous Ampicillin–Sulbactam three gram every eight hours and oral Azithromycin 250mg every 12 hours. The laboratory data are available in Table 1.

**TABLE 1:** Laboratory data

Test	Unit	First admission	Second admission	Discharge
WBC	*1000/mm3	6	6.1	7.3
segment	Percent	72	55.8	43.3
lymphocyte	Percent	19	39.2	42.1
Hb	g/dl	14.3	13.5	13.7
MCV	Fl	80.5	81.7	82.4
Plt	*1000/mm3	214	278	275
Creatinine	mg/dl	1.2	1	1.2
BUN	mg/dl	16	14	13
Na	mEq/dl	136	137	
K	mEq/dl	4.2	5.4	
P	mg/dl	3.5	3.8	
Ca	mg/dl	8.5	9.1	
Alb	g/dl	3.8		
Mg	mg/dl	1.9	2.7	
AST	IU/L	49	30	
ALT	IU/L	44	36	
Alk.p	IU/L	93	176	
Bili Total	mg/dl	0.4		
Bili Direct	mg/dl	0.2		
CPK	IU/L			
LDH	U/L		457	366
ESR	mm/hr	45	31	16
CRP	mg/l		6	6
PT	Sec	13	13	
INR	Indx	1	1	
PTT	Sec	39	29	

The nasopharyngeal swap was negative, but the serology of the COVID‐19 antibody for both IgM and IgG was positive. After 11 days of intravenous antibacterial therapy, the hemoptysis was resolved. At the follow‐up chest CT scan, the abscess was improving and showed signs of remission.

At discharge, oral Amoxicillin–Clavulanic acid 625mg every eight hours, supplemented with oral Ciprofloxacin 500mg every 12 hours, was prescribed for a week.

## 3 DISCUSSION

Even though COVID‐19 is a respiratory virus, research showed that this viral infection could involve many other organs in the body. Cardiovascular involvement, heart failure, renal failure, liver damage, shock, and multi‐organ failure are other manifestations' of COVID‐19.^6^ Studies even showed neurological complications of COVID‐19.^7^ The coronavirus can cause some hematologic complications such as lymphocytopenia, thrombocytopenia, coagulopathies, and disseminated intravenous coagulation.^8^

Lung abscess is usually a cavity formation as a result of lung tissue necrosis. Bacterial infection and aspiration can lead to abscess formation.

Lung tissue diseases cause primary abscesses. On the other hand, secondary abscesses are usually due to other medical conditions such as pulmonary thromboembolism or the spread of extrapulmonary abscesses to the lungs.

In this patient, the pulmonary symptoms and lung abscess started after discharge. The patient had no history of alcohol consumption or substance abuse. He showed no signs of esophageal dysfunction. He had not experienced any loss of consciousness or seizures. His oral hygiene was optimal. After precise history taking and laboratory data evaluations, immunodeficiency diseases were excluded. The only recent illness was COVID‐19 infection.

The patient was tested for tuberculosis, considering that tuberculosis is endemic in Iran. The results came back negative. Because of recent COVID‐19 infection and positive serology and clinical improvement of lung abscess after the administration of antibiotics, the treatment team decided that the bronchoscopy is not necessary for the patient.

To our knowledge, no previous studies have reported lung abscess as a complication of COVID‐19 infection. It is important to follow‐up with the patients after discharge for such complications, especially if the patients are symptomatic.

## 4 CONCLUSION

COVID‐19 is a novel virus, and a lot is remained to understand the pathophysiology of this disease. In this case report, we presented a previously healthy patient who developed a lung abscess after receiving treatment for COVID‐19. The practitioners should be aware of such complications and look for it.

## CONFLICT OF INTEREST

The authors declare that they have no conflict of interests.

## AUTHOR CONTRIBUTIONS

Dr. Nazanin Zamani gathers the information and prepares the manuscript. Dr. Oldooz ALoosh was the attending responsible for the treatment options in the second admission. Dr. Samane Ahsant was the fellow responsible for the treatment of the patient. Dr. Zeynab Yassin was the attending responsible for the treatment option during the first admission. Dr. Aminreza Abkhoo was the radiologist, consulting the treatment team. Dr. Taghi Riahi was the chief of the pulmonology department and in charge of the patient’s treatment plan.

## ETHICAL STATEMENT

For publishing this case report, we asked Rasoul‐Akram hospital ethical committee for approval. We informed the patient about the process of publishing a case report and he signed the consent form.

## Data Availability

The data that support the findings of this study are available on request from the corresponding author. The data are not publicly available due to the fact that their containing information that could compromise the privacy of research participants.
